# The *ACTwatch *project: methods to describe anti-malarial markets in seven countries

**DOI:** 10.1186/1475-2875-10-325

**Published:** 2011-10-31

**Authors:** Tanya Shewchuk, Kathryn A O'Connell, Catherine Goodman, Kara Hanson, Steven Chapman, Desmond Chavasse

**Affiliations:** 1 Malaria & Child Survival Department, Population Services International, P.O. Box 43640-00800, Nairobi, Kenya, Africa; 2 Department of Global Health and Development, London School of Hygiene and Tropical Medicine, 15-17 Tavistock Place, London WC1H 9SH, UK; 3 Population Services International, 1120 19th Street N.W., 20036, Washington D.C., USA

## Abstract

**Background:**

Policy makers, governments and donors are faced with an information gap when considering ways to improve access to artemisinin-based combination therapy (ACT) and malaria diagnostics including rapid diagnostic tests (RDTs). To help address some of these gaps, a five-year multi-country research project called *ACTwatch *was launched. The project is designed to provide a comprehensive picture of the anti-malarial market to inform national and international anti-malarial drug policy decision-making.

**Methods:**

The project is being conducted in seven malaria-endemic countries: Benin, Cambodia, the Democratic Republic of Congo, Madagascar, Nigeria, Uganda and Zambia from 2008 to 2012.

*ACTwatch *measures which anti-malarials are available, where they are available and at what price and who they are used by. These indicators are measured over time and across countries through three study components: outlet surveys, supply chain studies and household surveys. Nationally representative outlet surveys examine the market share of different anti-malarials passing through public facilities and private retail outlets. Supply chain research provides a picture of the supply chain serving drug outlets, and measures mark-ups at each supply chain level. On the demand side, nationally representative household surveys capture treatment seeking patterns and use of anti-malarial drugs, as well as respondent knowledge of anti-malarials.

**Discussion:**

The research project provides findings on both the demand and supply side determinants of anti-malarial access. There are four key features of *ACTwatch*. First is the overlap of the three study components where nationally representative data are collected over similar periods, using a common sampling approach. A second feature is the number and diversity of countries that are studied which allows for cross-country comparisons. Another distinguishing feature is its ability to measure trends over time. Finally, the project aims to disseminate findings widely for decision-making.

**Conclusions:**

ACTwatch is a unique multi-country research project that threads together anti-malarial supply and consumer behaviour to provide an evidence base to policy makers that can help determine where interventions may positively impact access to and use of quality-assured ACT and RDTs. Because of its ability to detect change over time, it is well suited to monitor the effects of policy or intervention developments in a country.

## Background

Artemisinin-based combination therapy (ACT) is recommended by the WHO as the first-line treatment of *Plasmodium falciparum *malaria. By 2006, most malaria endemic countries had changed their national treatment guidelines to follow these recommendations [1], but despite increased financing for malaria control over the last decade [2], the global target of at least 80% of children under five with malaria receiving an effective anti-malarial is far from being met [3]. Today, the use of ineffective monotherapies remains widespread [4] and the development of artemisinin resistance [5] has raised specific concerns around how to reduce the use of artemisinin monotherapies in order to contain resistance to this drug that is largely seen as the only effective treatment option currently available [6].

Many countries are accelerating their efforts to increase coverage of ACT, with support from funders including the Global Fund to Fight AIDS, Tuberculosis and Malaria, the President's Malaria Initiative and the World Bank Booster Programme. In 2010 alone, more than 200 million doses of ACT were forecasted to enter the market [7]. The most significant recent intervention is the Affordable Medicines Facility-malaria (AMFm) launched in 2010 that aims to increase access to high-quality ACT in the public and private sectors through a novel co-payment fund worth $216 million in Phase 1 [8]. To date, orders for over 150 million treatments have been placed mainly by the private sector in the 9 pilot programmes operating in eight countries [9].

Despite these and other on-going efforts to increase access, robust evidence on the availability and use of the different anti-malarials is sparse. Policy makers, governments and donors are faced with an information gap when trying to determine how to improve access to high quality ACT and reduce the use of artemisinin monotherapies; and rigorous approaches are needed to evaluate interventions such as the AMFm. [10]. Furthermore, since WHO issued the recommendation that anti-malarials be restricted to patients with a confirmed malaria diagnosis [11], information on the availability of diagnostic services and tools such as RDTs in the market has become crucial.

To help address some of these gaps, Population Services International (PSI) in partnership with the London School of Hygiene and Tropical Medicine (LSHTM) launched a five-year multi-country research project in 2008 called *ACTwatch*. The project aims to provide a comprehensive picture of the anti-malarial market to inform national and international policy makers. It is designed to detect changes in the availability, price and use of anti-malarials over time and between sectors, and to monitor the effects of policy or intervention developments at country level. *ACTwatch *addresses both the supply and demand sides of the market. The supply side is evaluated by collecting data in public and private sector outlets and wholesalers of anti-malarial drugs. To evaluate demand, data on consumer treatment-seeking behaviour and knowledge are collected at the household level. In combination, the research components thread together the anti-malarial market and consumer behaviour to provide this comprehensive overview. Findings can help determine where and to what extent interventions have positively impacted access to and use of quality-assured ACT and RDTs as well as inform artemisinin resistance containment efforts.

## Methods

An *ACTwatch *Advisory Committee was established to help ensure that the project's methods were rigorous and its outputs relevant to policy makers. The committee is made up of 19 leading professionals comprising academics, researchers, public health implementers, donors, advocates and members from the private sector. In addition, consultation was held with the respective national malaria control programmes and other relevant bodies. Necessary authorizations including ethical approval were sought and obtained for each study country.

### Population

**Figure 1:**
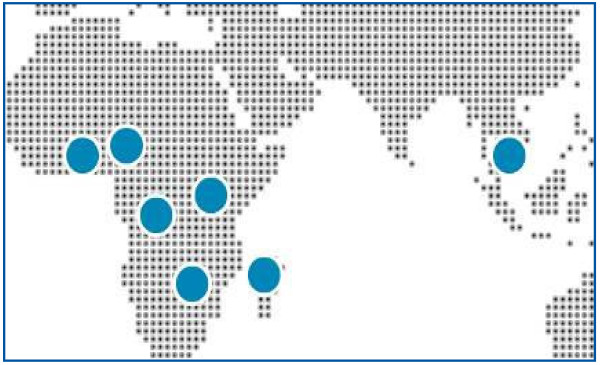
**Map of countries included in the project**.

The project is being conducted in seven malaria-endemic countries: Benin, Cambodia, the Democratic Republic of Congo, Madagascar, Nigeria, Uganda and Zambia between 2008 and 2012 (Figure 1). Countries were selected with the aim of studying a diverse range of markets from which comparisons and contrasts could be made considering factors such as demand for anti-malarials (reflected by malaria burden), size of the population at risk, pharmaceutical regulation (high/low), nature of pharmaceutical regulation (Francophone versus Anglophone), public sector capacity and coverage, existence of local anti-malarial manufacturing, existence of anti-malarial subsidy interventions and the feasibility of receiving necessary country level authorization to conduct the research.

### The research questions and indicators

The research sets out to measure which anti-malarials are available where and at what price, and who they are used by. It examines market share of different anti-malarials passing through public facilities and private retail outlets. It also provides a picture of the distribution channels serving these outlets, mapping out the supply chain and the mark-ups levied across each level. Provider knowledge and attitudes are also assessed. On the demand side, *ACTwatch *measures treatment seeking patterns and use of anti-malarial drugs, as well as respondent knowledge and awareness of ACT at the household level. It also explores factors that influence treatment choices. Further details can be found in accompanying publications (O'Connell *et al*: Got ACTs? Availability, price, market share and provider knowledge of antimalarial medicines in public and private sector outlets in six malaria-endemic countries, submitted) and Littrell *et al: *Monitoring fever treatment behavior and equitable access to effective medicines in the context of initiatives to improve ACT access: baseline results and implications for programming in six African countries, submitted).

**Figure 2:**
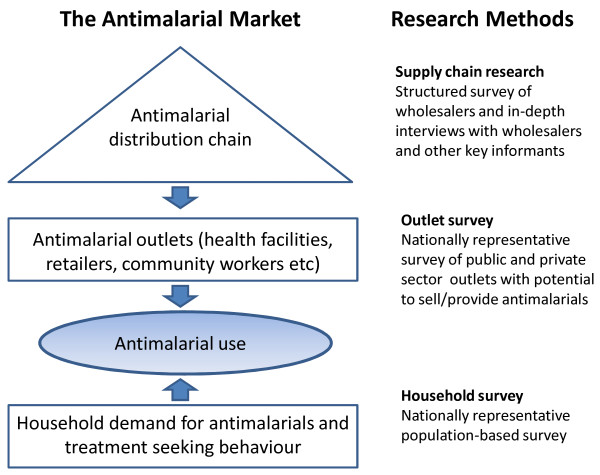
**Research Methods to Study Supply and Demand**.

Three different studies are conducted in each country, as shown in Figure 2, which together provide a comprehensive understanding of the anti-malarial market: 1) an outlet survey gathering information on availability; 2) a household survey in the same geographical areas assessing treatment-seeking behaviour, usage and purchase price and 3) a supply chain mapping including an analysis of mark-up at each level. All components are conducted as close in time as practicable in order to match supply and demand side data as well as to allow for triangulation of the results.

The indicators used for each study component were developed in consultation with partners and the Advisory Committee and are, as far as possible, consistent with those proposed for the independent evaluation of AMFm [12], Demographic and Health Surveys [13] and Malaria Indicators Surveys [14]. Table 1 illustrates the project's key indicators.

**Table 1:** Selected Project Indicators

Category	Indicator description
Availability	*Proportion of outlets that:*
	• Have in stock: any anti-malarial, specified anti-malarial categories
	• Reported no disruption in stock of anti-malarials in the last three months
	• Have either rapid diagnostic tests or microscopic blood testing facilities

Price	• Median price of a full adult course by anti-malarial category
	• Household price of fever episode treated with an anti-malarial

Market Share	• Total volume of an anti-malarial category sold or distributed in the last week as a proportion of the total volume of all anti-malarials sold or distributed in the last week

Provider/wholesaler knowledge and behaviour	*Proportion of providers that:*
	• Correctly state: the recommended first line treatment, the correct dosing regimen
	• Can list at least one danger sign in a child that requires referral to a public health facility

Use	*Proportion of children under five with fever in the past 2 weeks that:*
	• Received anti-malarial treatment, by anti-malarial category, and source, the same or next of fever onset, by socio-economic quintile
	• Received a blood test for malaria the same or next day of fever onset

Caregiver knowledge and behaviour	*Proportion of Caregivers that:*
	• Sought treatment for fever, by source, and reason(s) for this choice(s)
	• Can name the first-line drug. Know a common brand of first-line ACT.
	• Request a specific anti-malarial by category or brand name from providers.
	• Express their opinion on the most effective malaria treatment for adults, by anti-malarial category

Supply chain structure	• Minimum and maximum number of steps in the distribution chain
	• Proportion of wholesalers that engage in specified business practices
	• Proportion of wholesalers that have specific categories of anti-malarials and RDTs in stock
	• Median percent and absolute mark-up applied by wholesalers, and retailers, by anti-malarial category

### The outlet survey

The outlet survey studies both the public and private sectors in order to have a complete picture of the anti-malarial market within a country at the retail level. The survey is conducted approximately three times over the life of the project in each country to measure trends over time.

A cross-sectional survey is conducted of outlets with the potential to stock and dispense anti-malarials to patients/caregivers. Building on previous outlet survey methodologies [15-17], the outlet survey measures price, availability and volumes of anti-malarial medicines and the price and availability RDTs. The sample is based on a one-staged cluster design using probability proportion to population size, that provides nationally representative data sufficiently powered to allow for comparisons over time, between the public and private sectors and across sub-populations where stratified. The sampling strategy was designed to detect a 20 percentage point change in the primary outcome measure, availability of ACT. The cluster selected was an administrative unit with on average 10, 000 to 15, 000 inhabitants, such as a sub-district or parish.

Stratification was determined by consulting with the Ministry of Health (MOH) in each country to identify characteristics of sub-populations that are relevant for policy consideration. Table 2 provides an overview of country stratification, which varies across countries by number and characteristics.

**Table 2:** Country Stratification

Country	# of Strata	Strata Description
Benin	1	None

Cambodia	2	1. Areas where *P. falciparum *parasite resistance to artemisinin is documented ("Containment Zone 1") or where resistance is feared to have spread but not formally detected ("Containment Zone 2") 2. Other Areas of the country *Source: Cambodia National Malaria (CNM) Programme, Kingdom of Cambodia*

Democratic Republic of Congo (DRC)	4	1. North-East Provinces: Oriental, Nord Kivu, Sud Kivu, and Maniema 2. North West Provinces: Bas-Congo, Bandundu, and Equateur 3. Centre-South Provinces: Katanga, Kasai Oriental, and Kasai Occidental 4. Capital city: Kinshasa *Source: Localized censuses in health zones conducted between 2001 and 2004, with the support of various INGOS*

Madagascar	2	1. Urban areas 2. Rural areas *Source: Cartographie censitaire de la population 2009. Institut National de Statistique*.

Nigeria	6	1. North-Central states: Benue, Abuja-FCT, Kogi, Kwara, Nasarawa, Niger, Plateau 2. North East states: Adamawa, Bauchi, Borno, Gombe, Taraba, Yobe 3. North West states: Jigawa, Kaduna, Kano, Katsina, kebbi, Sokoto, Zamfara 4. South East states: Abia, Anambra, Ebonyi, Enugu, Imo 3. South South states: Akwa ibom, Bayelsa, Cross rivers, Delta, Edo, Rivers 4. South West states: Ekiti, Lagos, Ogun, Ondo, Osun, Oyo *Source: Nigeria National Population Commission*

Uganda	2	1. Low malaria transmission areas 2. Medium to high malaria transmission areas *Source: Malaria Control Programme, Ministry of Health, Uganda*

Zambia	2	1. Urban areas 2. Rural areas *Source: Zambia Population Census Frame, 2000. Central Statistical Office, Lusaka, Zambia*

In order to capture the market as a whole rather than some of its segments, all outlet types with the potential to dispense anti-malarials were included and a census of these within each selected cluster was conducted. Outlets with this potential were identified at a country level through consultation with local stakeholders. The types of outlets vary somewhat from country to country, and are classified as falling either within the Public/Not-for-Profit Sector or the Private Sector categories. The Public/Not-for-Profit sector consists of public health facilities, community health workers and not-for-profit health facilities such as mission and NGO-supported facilities. The Private Sector is made up of private health facilities and pharmaceutical outlets authorized to sell prescription medicines, and a diverse range of other providers with fewer or no health qualifications, such as drugs stores, grocery stores, street hawkers and kiosks.

**Figure 3:**
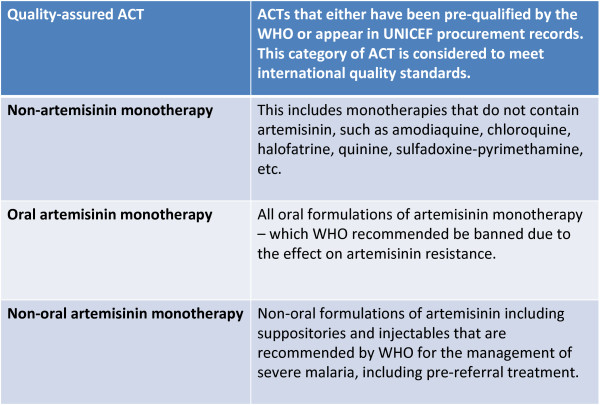
**Presenting results: Classifying anti-malarial drugs**.

**Figure 4:**
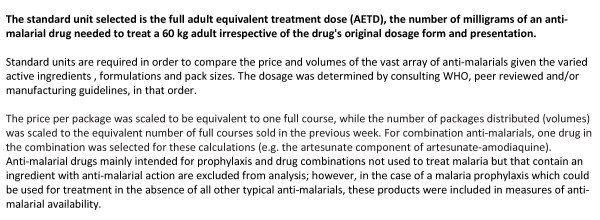
**Presenting results: Employing standard units**.

All anti-malarials found within an outlet are captured in the survey. This means that drugs are captured regardless of whether they are registered in the country or recommended by the WHO. Given the large numbers of different anti-malarials available on the market, anti-malarials captured through the surveys are classified into policy relevant categories when presenting results. The classification of anti-malarials for analysis is shown in Figure 3 along with a description of standard unit employed, the adult equivalent treatment dose (AETD) in Figure 4. Outlet Survey methods and results are presented in full in O'Connell *et al *(submitted).

### The household survey

The objective of the household survey component is to monitor consumer treatment-seeking behaviour for fever. It is a population based, cross-sectional survey that takes place twice over the length of the project, at baseline and end-line, either at the same time or soon after the collection of outlet survey data. It takes place in the same geographical areas as the outlet survey and allows for comparisons over time and across strata, where relevant. It is powered to detect a 20 percentage point difference in the key outcome indicator "the proportion of children under five with fever in the past two weeks who used any anti-malarial the same or next day of the onset of fever".

The study mirrors methods used in typical population based surveys and follows standard Demographic and Health Survey sampling procedures. A household is eligible if there is a child under 5 with a history of fever in the past two weeks, except in Cambodia, where eligibility is based on any family member with fever in the past two weeks due to low malaria prevalence rates. The questionnaire captures information on the type of anti-malarial treatments taken (if any), the price paid for treatment and related fees, treatment sources, distance travelled to obtain treatment, knowledge related to malaria, any diagnostic test received and price of diagnosis. An additional module was also administered to adults in an attempt to ascertain types of anti-malarial treatment taken for fever, source and price, which could be matched with supply side results. Formative research was conducted at the country level in order to identify potential determinants of appropriate treatment behaviour. These were subsequently included in the main survey as multi-item scales aimed at capturing attitudes and perceptions of respondents, which assist with identifying information on determinants needed for demand creation activities. Household Survey methods and results are presented in full in Littrell *et al*(submitted).

### The supply chain study

The supply chain component employs quantitative and qualitative methods to study the distribution chain for anti-malarial drugs from factory gate/port of entry to consumer, mark-ups along the supply chain, market structure including level and forms of competition, and the policy/regulatory environment. The objective is to provide policy makers with a map of the supply chain for anti-malarials and RDTs, including evidence on wholesaler volumes and the components of the consumer price in the context of the current market and policy influences on the supply chain. Data collection methods include a structured survey of wholesalers of anti-malarials and qualitative in-depth interviews throughout the distribution chain.

We conduct a cross-sectional survey amongst wholesalers that operate in the private commercial distribution chain that serves the public and private sector. Respondents are identified through a bottom-up approach during which wholesalers are identified by their customers until the top of the chain is reached. The survey collects data on the supply chain structure, wholesaler characteristics and business practices, wholesale outlet licensing and inspection, wholesaler knowledge, qualifications and training, and availability, purchase price and mark-ups for anti-malarials and RDTs.

A sub-set of retailers and wholesalers in the distribution chain undergoes in-depth interviews which collect data on questions that are not readily addressed using quantitative methods. This includes complex issues such as horizontal and vertical integration of the market; subjective perceptions and opinions of staff; and the exploration of sensitive commercial and regulatory issues.

Supply chain research methods are presented in full in "Supply Chain Survey Results, Cambodia, January 2011" [18].

## Dissemination

An additional objective of *ACTwatch *is to ensure that policy makers at both the national and international level have access to our findings through dissemination. At the national level, we organize workshops targeting key stakeholders such as donors, government, and NGOs. The workshop serves as an opportunity for country-level stakeholders to interpret and discuss results in the context of their operating environment. Baseline results discussed by O'Connell *et al *(submitted) and Littrell *et al *(submitted)have been presented to stakeholders in ACTWatch countries. Dissemination at the international level is conducted at scientific conferences (MIM 2009, ASTMH, IHEA) and meetings targeting policy makers including donors, such as PMI and the Global Fund, and international organizations. The advisory committee has helped shape the presentation of results, particularly multi-country comparisons, in order to convey their relevance. Country-specific results as well as study designs and tools are available online [19].

## Discussion

The past decade has seen important investments to increase access to quality-assured ACT. However, policy makers have had limited evidence on which to draw in selecting among different delivery channels. On the supply side, research has tended to focus on the public sector's health facilities and supply chain rather than the market as a whole, with some limited exceptions [20,21]. On the demand side, large-scale surveys including DHS, MICS and MIS, have generally been conducted and interpreted in the absence of an understanding of the overall market.

The *ACTwatch *research project presents an opportunity to provide policy-makers with results on both the demand and supply side determinants of anti-malarial access. There are four key features of this project. First is overlap of the outlet, household and supply chain components where nationally representative data are collected over similar periods and using a common sampling approach. This allows for a better understanding of how variables such as availability and price collected through the outlet survey may affect the treatment decisions collected during the household survey; or how mark-ups at different levels of the supply chain affect final retail prices and household costs. A second feature is the number and diversity of countries that are captured using comparable methods, including standardized tools, intensive training of field staff, rigorous monitoring procedures and centralized analysis. The approach allows for results to be compared across countries and should fuel the international policy debate. It is anticipated that policy-makers will be as interested in common themes that run across countries as they will of real differences between countries. Another distinguishing feature of this research is its ability to measure trends in nationally representative data over time. A final feature is the project's aim to disseminate findings widely so that policy-makers may use these for decision-making in specific areas such as drug regulation, supply chain management and training packages as well as for higher level policies such as the role of the private sector in the anti-malarial market.

These strengths make the research design well adapted to monitor the impact of the AMFm pilot on drug prices, market share, availability and use. Four of the eight AMFm phase 1 pilot countries are included in the *ACTwatch *project: Madagascar, Nigeria, Uganda and Cambodia, and the indicators that we collect overlap with those being employed by an independent evaluation commissioned as part of the AMFm's Monitoring & Evaluation Technical Framework. Our outlet survey methodologies, tools and results have fed into the study design developed by the Independent Evaluator and relevant country outlet survey results collected by *ACTwatch *will form part of the evaluation's baseline and endline data. In addition, early *ACTwatch *supply chain data and outlet survey results were employed in designing AMFm country proposals suggesting that our results could be useful for planning other implementation strategies. It is also likely that comparisons between the results of AMFm and non-AMFm countries captured in our research will be made.

Despite its strengths, the research methodology has several limitations. First, given the observational study design, it may be difficult to identify the influence of individual factors on trends observed over time, as many factors such as changes in donor funding, regulation and other interventions may simultaneously affect our key outcomes in a given period. Second, given the cross-sectional nature of the surveys, data are only collected over a relatively short time period and do not capture seasonal and other fluctuations in supply and demand. However, as the outlet and household surveys are conducted at a national level during malaria transmission periods, the data can be considered to provide an accurate picture of the situation during key seasons for malaria treatment. Third, data collected are respondent-dependent and the respondent could withhold or misrepresent some information. On the supply side in particular, providers and wholesalers may prefer not to provide accurate information on sensitive topics such as mark-ups or stocking rates of substandard products, particularly in countries where there is a strong regulatory structure. That said, certain data such as types of drugs and price can be triangulated between wholesaler, outlet and household levels to check accuracy of findings.

Another challenge is related to ensuring that the research has country-specific relevance without compromising the ability to compare across countries. Although standardized, the methodology is sufficiently supple to capture sub-population stratification requirements with adequate power, and differences in market structure and outlet types for each country. The challenge that this adaptability has posed is in determining how far changes can be made without jeopardizing comparability of results and has meant that the research is not always as country-relevant as local stakeholders would like. This is an intrinsic problem to multi-country studies, which in this case is heightened by the diversity of the selected study countries.

A key question is whether it is feasible to continue to research anti-malarial markets on such a large scale on an on-going basis. One option would be to use existing national household surveys such as the DHS to provide information on the use of anti-malarials. One of the drawbacks to such an approach is a risk that temporal proximity across study components could be lost as timelines would no longer be within the control of this research project. It is also possible that these existing surveys may not be in position to collect a sufficient level of detail. There are no other sources of standardized supply side data (outlet or supply chain).

## Conclusion

*ACTwatch *is a unique multi-country research project that sheds light on the complicated and often unregulated anti-malarial market by threading together anti-malarial supply and consumer behaviour. It provides countries with essential information necessary to define interventions to increase access to recommended quality-assured ACT and RDTs. In addition, because of its ability to detect change over time, it is well suited to monitor the effects of donor investments and novel mechanisms like the AMFm.

## Competing interests

The authors declare that they have no competing interests.

## Authors' contributions

KOC and KH are co principal investigators. CG, KOC, SC, and KH contributed to the development of research methods. DC and SC conceived the project. TS provides project oversight. TS drafted the manuscript and all authors provided critical review. All authors read and approved the final manuscript.
